# Multidisciplinary team in temporomandibular disorders

**DOI:** 10.1080/07853890.2021.1897363

**Published:** 2021-09-28

**Authors:** Pedro Cebola, Paula Moleirinho-Alves, André Mariz de Almeida

**Affiliations:** a Instituto Universitário Egas Moniz (IUEM), Egas Moniz Cooperativa de Ensino Superior, Caparica, Portugal; b Escola Superior de Saúde Egas Moniz, Caparica, Portugal; c Centro de Investigação Interdisciplinar Egas Moniz (CiiEM), Egas Moniz Cooperativa de Ensino Superior, Caparica, Portugal

## Abstract

**Introduction:**

The temporomandibular disorders (TMD) are one of the main concerns regarding orofacial pathologies and there are an ascending number of cases. They are characterised as a group of pathological conditions that may affect the temporomandibular joint (TMJ), the masticatory musculature and/or other adjacent anatomical structures, leading to pain and dysfunction [1]. The multifactorial aetiology of TMD affects a relatively large number of the world population and requires a multidisciplinary evaluation and diagnosis by the clinical team [2]. Among the various elements that constitute the multidisciplinary team we highlight the dentist and the physiotherapist. The dentist as a first-line professional, most of the time is responsible for the identification of patients potentially at risk and for the follow-up of those who already present the disease [3]. On the other hand, the physiotherapist aims to reduce musculoskeletal pain, promote muscle relaxation, reduce muscle hyperactivity, improve function by restoring the quality and quantity of mandibular movements and maximise joint mobility [4].

**Description of the clinical case:**

A 24-year-old female patient with a history of temporomandibular disorder and bruxism she presented an non-assisted mouth opening (UMO) of 28 mm and an assisted mouth opening with pain in the masseter and TMJ bilaterally of 29 mm, after the clinical assessment with the DC/TMD protocol we arrived at a diagnosis of disc displacement without reduction (DDwR) with limited opening, degenerative joint disease in the left TMJ, arthralgia (II), myofascial pain (III) in the masseter with pain referred to other anatomical regions. All the assumptions of the Helsinki Declaration have been fulfilled and an informed consent for clinical case of Clinica Dentária Egas Moniz approved by the ethic commission of Instituto Universitário Egas Moniz. The treatment plan consisted of cognitive behavioural therapy (CBT), prescription of muscle relaxants, occlusal splint especially for reduction of overload due to bruxism, infiltration with 1 ml of hyaluronic acid of high molecular weight in the TMJ bilaterally followed by articular mobilisation techniques and neuromuscular and myofascial techniques. The patient was instructed to continue with the physiotherapy.

**Results:**

The follow up was made 2 months after with 8 sessions of physiotherapy and 1 more hyaluronic acid infiltration bilaterally as protocoled session, we could observe an absence of temporomandibular joint pain, UMO of 42 mm, decreased crepitation, decreased intensity of myofascial pain (I) and (DDwR) without limited opening.

**Discussion and conclusion:**

The multidisciplinary dentist-physiotherapist team combines inputs from different professions with the aim of promoting the best patient care and represents an added value for the management of the signs/symptoms of patients with temporomandibular dysfunction.
